# Can Bone Tissue Engineering Contribute to Therapy Concepts after Resection of Musculoskeletal Sarcoma?

**DOI:** 10.1155/2013/153640

**Published:** 2013-01-14

**Authors:** Boris Michael Holzapfel, Mohit Prashant Chhaya, Ferry Petrus Wilhelmus Melchels, Nina Pauline Holzapfel, Peter Michael Prodinger, Ruediger von Eisenhart-Rothe, Martijn van Griensven, Jan-Thorsten Schantz, Maximilian Rudert, Dietmar Werner Hutmacher

**Affiliations:** ^1^Regenerative Medicine Group, Institute of Health and Biomedical Innovation, Queensland University of Technology, 60 Musk Avenue, Kelvin Grove, QLD 4049, Australia; ^2^Department of Orthopaedic Surgery, König-Ludwig-Haus, Orthopaedic Center for Musculoskeletal Research, University of Würzburg, Brettreich Straße 11, 97072 Würzburg, Germany; ^3^Department of Orthopaedics, University Medical Center Utrecht, Heidelberglaan 100, 3584 CX Utrecht, The Netherlands; ^4^Department of Orthopaedic Surgery, Klinikum Rechts der Isar, Technical University Munich, Ismaninger Straße 22, 81675 Munich, Germany; ^5^Experimental Trauma Surgery, Department of Trauma Surgery, Klinikum Rechts der Isar, Technical University Munich, Ismaninger Straße 22, 81675 Munich, Germany; ^6^Department of Plastic Surgery and Hand Surgery, Klinikum Rechts der Isar, Technical University Munich, Ismaninger Straße 22, 81675 Munich, Germany; ^7^The George W. Woodruff School of Mechanical Engineering, Georgia Institute of Technology, 801 Ferst Drive Northwest, Atlanta, GA 30332, USA

## Abstract

Resection of musculoskeletal sarcoma can result in large bone defects where regeneration is needed in a quantity far beyond the normal potential of self-healing. In many cases, these defects exhibit a limited intrinsic regenerative potential due to an adjuvant therapeutic regimen, seroma, or infection. Therefore, reconstruction of these defects is still one of the most demanding procedures in orthopaedic surgery. The constraints of common treatment strategies have triggered a need for new therapeutic concepts to design and engineer unparalleled structural and functioning bone grafts. To satisfy the need for long-term repair and good clinical outcome, a paradigm shift is needed from methods to replace tissues with inert medical devices to more biological approaches that focus on the repair and reconstruction of tissue structure and function. It is within this context that the field of bone tissue engineering can offer solutions to be implemented into surgical therapy concepts after resection of bone and soft tissue sarcoma. In this paper we will discuss the implementation of tissue engineering concepts into the clinical field of orthopaedic oncology.

## 1. Introduction

Bone has to carry major loads. To fulfil this task it is created as a composite material, which comprises primarily of collagen, noncollageneous proteins, and hydroxyapatite. Its complex structure contains a wealth of mechanically relevant details [1]. Bone is a composite in several senses that is, being a porous material, a polymer-ceramic mixture, a lamellar material, and a fibre-matrix material. Its mechanical properties will therefore depend on each of these aspects of composition and structure. In general, bone displays a high intrinsic regenerative capacity following trauma or disease. Therefore, the majority of fractures heal spontaneously by a recapitulation of the pathway of normal fetal skeletogenesis, including endochondral and intramembraneous ossification [4]. Refinements in surgical techniques, implant design and postoperative care have significantly improved treatment outcomes of complex fractures and defects as caused by high-energy trauma, disease, developmental deformity, and revision surgery. However, there are conditions in which bone regeneration is compromised or in which bone regeneration is required in a large quantity. 

A situation of the latter entity is the resection of malignant bone and soft tissue sarcoma. This can result in large defects where regeneration is needed in a quantity far beyond the normal potential of self-healing [5]. Furthermore, an adjuvant therapeutic regimen or local factors such as postoperative seroma or infection can account for a limited intrinsic regenerative potential [6]. Therefore, reconstruction of these defects is still one of the most demanding procedures in orthopaedic surgery.

## 2. Common Treatment Strategies for Bone Defects 

Treatment protocols of bone and soft tissue sarcoma are based not only on the tumour biology and location, but also on the patient's needs and age [7]. The primary surgical goal should be to obtain adequate surgical margins in order to ensure local tumor control [8–10]. With the introduction of multimodal therapeutic concepts and improved reconstruction techniques, limb salvage procedures have largely replaced ablative surgery [11–14], but only few of them can restore the original anatomical and functional conditions. For the reconstruction of skeletal defects after tumor resection, both biological techniques (e.g., autografts, allografts, or rotationplasty) and prostheses are used. These procedures, mainly bone grafting and metallic implants, are well established and the comparative advantages and disadvantages have been discussed at length in the literature [15–17]. Problems of autologous bone grafting can be donor site morbidity and limitation of the graft mass. For graft harvesting, additional personnel and time are needed. The use of allografts or xenografts carries the risk of immunomediated rejection, transmission of infectious diseases, or graft sequestration. In addition, the acquisition costs of allo- or xenografts are rather high. Graft devitalisation and consecutive absorption processes can lead to decreased mechanical stability. Failures usually result from incomplete transplant integration, particularly in critical sized defects. Due to the dense nature of cortical allografts, revascularisation and cellular invasion is impeded. This limited ability for revascularization and remodelling is believed to be responsible for the high complication rate associated with allografts [18]. Other biologic approaches used for the reconstruction of bone defects include distraction osteogenesis, segment transport, or the Masquelet technique, but all of these methods are technically demanding and they require lengthy treatment protocols, which can be highly inconvenient for patients [19, 20]. The limitations of these conventional biological reconstruction techniques are exacerbated in cancer patients, who are often elderly, have localised or systemic osteoporosis and suffer from impaired wound healing as a consequence of an adjuvant therapeutic regimen. The high tensile strength and fatigue resistance of metal would make it suitable for load-bearing applications, but the large mismatch in Young's modulus between metal and bone can lead to peri-implant bone resorption, a phenomenon known as stress shielding [21]. Furthermore, tumor endoprostheses exhibit a higher complication rate than standard implants with infection or aseptic loosening as the most common failure modes [22].

These constraints have triggered a need for new therapeutic concepts to design and engineer unparalleled structural and functioning bone grafts to replace current treatment options. To satisfy the need for long-term repair and good clinical outcome, a paradigm shift is needed from methods to replace tissues with inert medical devices to more biological approaches that focus on the repair and reconstruction of tissue structure and function [23]. It is within this context that the field of bone tissue engineering can offer solutions to be implemented into surgical therapy concepts after resection of bone and soft tissue sarcoma. While this has already led to a variety of novel therapeutic concepts particularly in the field of craniofacial surgery [3, 24], only few called smart biomaterials have found their way into clinical application in the field of orthopaedic surgery. 

In the following passages, we will discuss the implementation of tissue engineering concepts into treatment strategies of bone defects caused by musculoskeletal sarcoma. From a material science and especially clinical point of view, the future prospects and possible application spectrum will be outlined. 

## 3. Tissue Engineering Constructs (TECs)

The field of tissue engineering is embodied in the collective vision of its early pioneers Langer and Vacanti, whose diverse yet symbiotic research approaches as an engineer and surgeon led to the commencement of this interdisciplinary field. Their seminal 1993 paper remains one of the most influential and cited works in the field [25]. The application of the principles of biology and engineering to the development of functional substitutes for damaged tissue has seen laboratories worldwide forging impressive multidisciplinary teams to focus on restoring, maintaining, or improving the function of a wide range of human tissues. While progress has been made to deliver bench to bedside solutions, the rate at which tissue engineering has seen innovations translated to the clinic has been slower than originally expected and the urgency for tissue-engineered products which achieve these ideals remains high [26–28]. 

The fundamental concept underlying tissue engineering is to combine a scaffold with living cells and/or biologically active molecules to form a “tissue engineering construct” (TEC) which promotes the repair and/or regeneration of tissues [29, 30]. A suitable scaffold should (i) possess a porous interconnected pore network (pores and pore interconnections should be at least 400 *μ*m to allow vascularisation) with surface properties optimised for the attachment, migration, proliferation, and differentiation of cell types of interest (depending on the targeted tissue) and enable flow transport of nutrients and metabolic waste, (ii) be biocompatible, and (iii) be biodegradable with a controllable rate to complement cell/tissue growth and maturation [23, 31]. The design of these scaffolds also needs to consider physicochemical properties and morphology. External size and shape of the construct are of importance, particularly if the construct is customised for an individual patient. The work by groups focussing on scaffold design and fabrication utilising additive manufacturing technologies has advanced the tissue engineering field tremendously over the past few years [32]. The ability to create scaffolds in a layer-by-layer manner enables a computer-aided design to be directly translated from a clinical scan (i.e., a patient CT or MRI scan) to produce customised and/or patient-specific scaffolds to fit any anatomical defect site [33, 34]. 

## 4. Regeneration and Remodelling of TECs

**Figure 1:**
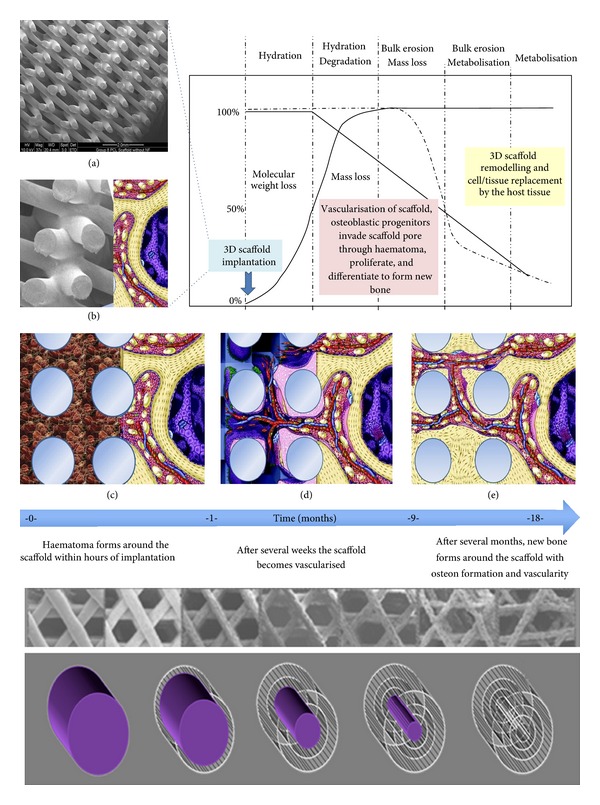
Schematic illustrating the interdependence of molecular weight loss and mass loss of a slow-degrading composite scaffold plotted against time. Scaffold implanted at *t* = 0 with lower figures showing a conceptual illustration of the biological processes of bone formation over time. After implantation the scaffold is immediately filled with haematoma followed by vascularisation. New bone is formed gradually within the scaffold. As the scaffold degrades over time, there is increased bone remodelling within the implant site until the scaffold pores are entirely filled with functional bone and vascularity (partially adapted from [2]). The lower part of the figure shows the schematic visualisation of how medical-grade poly-*ɛ*-caprolactone/tricalcium phosphate (mPCL-TCP) degrades via long-term bioerosion processes.

After scaffold implantation, continuous cell and tissue remodelling is essential to achieve and maintain stable biomechanical conditions, vascularization, and integration within the host site [2]. Importantly, TECs should stimulate and support both the onset and the continuance of bone ingrowth as well as subsequent remodelling and maturation by providing optimal stiffness and external and internal geometrical shapes. Scaffolds must provide sufficient initial mechanical strength and stiffness to substitute for the loss of mechanical function of the diseased, damaged, or missing tissue. Furthermore TECs must degrade at a rate which is compatible with new tissue ingrowth and maturation [35]. It is essential to understand and control this scaffold degradation process for successful tissue formation, remodelling and maturation at the defect site. In the early days of tissue engineering, it was believed that scaffolds should degrade and vanish as the tissue is growing [36]. Though, tissue ingrowth and maturation differ temporally from tissue to tissue and, furthermore, tissue ingrowth does not equate to tissue maturation and remodelling. In other words, a defect filled with an immature tissue should not be considered as “regenerated.” Hence, many scaffold-based strategies have failed in the past as scaffold degradation was more rapid than tissue remodelling and/or maturation [37]. Our concept of using a slow degrading composite scaffold fabricated with pores and pore interconnections with a size larger than 400 *μ*m is illustrated in Figure 1.

## 5. Translating Bone Tissue Engineering Concepts into the Clinical Field of Orthopaedic Oncology

Bone defects after resection of musculoskeletal tumours represent a considerable surgical challenge, are associated with high socioeconomic costs and highly influence patients' quality of life. These problems may be approached from the perspective of the nature of the graft material with which the surgeon works. The mission of our interdisciplinary group is to coordinate efforts between researchers and clinicians in the area of bone tissue engineering and the translation of tissue engineering platforms into orthopaedic oncology. Laboratories in Singapore, Germany, and Australia have spent the last decade in close collaboration translating a concept of bone tissue engineering based on slowly biodegradable composite scaffolds comprising medical grade poly (epsilon-caprolactone) (mPCL) and calcium phosphates from bench to bedside [38–45]. After a large series of *in vitro* experiments, we consequently performed small animal studies using mouse, rat, and rabbit models which demonstrated the ability of composite scaffolds in combination with BMPs or cells to promote bone regeneration within ectopic sites or bone defects [35]. Another key project of our group has been the development of a large animal model for bone regeneration research. We recently have established and fully characterised a critically sized defect model in sheep tibiae to evaluate different tissue-engineering-based treatment strategies [46, 47]. 

In the following section, we will describe a part of the rationale and road map of how our multidisciplinary research team is approaching the first steps to translate bone tissue engineering concepts into orthopaedic oncology. 

**Figure 2:**
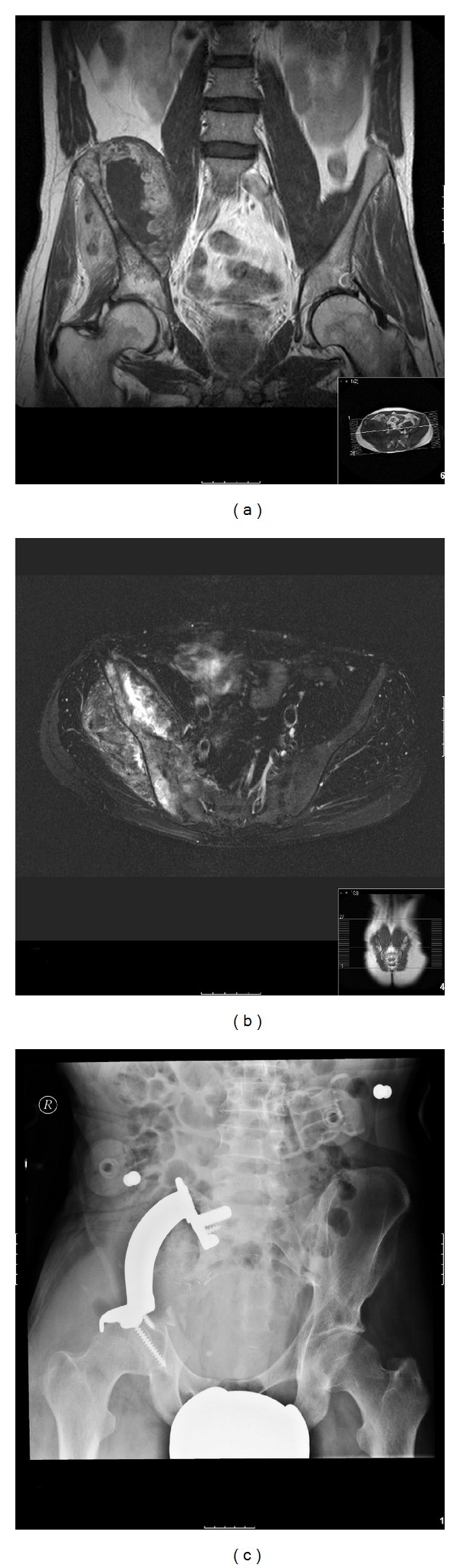
34 year old patient with a large destructive soft tissue mass of the right hemipelvis. Coronal T1-weighted (a) and axial T2-weighted (b) MRI images demonstrate calcific lobules and punctuated foci with low signal intensity representing calcifications, which are typical for chondroid matrix. Histological analysis revealed a dedifferentiated chondrosarcoma. After resection of the affected bone a custom-made pelvic metal prosthesis was fitted into the defect. Radiograph one year postoperatively (c) shows a stable prosthesis, the functional and clinical outcome of the patient was good.

Our clinical partners have used custom-made metal prostheses for the treatment of pelvic defects after sarcoma resection for more than 20 years [48, 49]. Since 1988, the general production process of the prosthesis has only changed in details but has developed according to the technological advances available. In the first step a 1 : 1 pelvic model is made using data acquired via high-resolution computed tomography. The model is cut out from a block of polyurethane by a five axial CNC-milling machine. In the next step the surgeon uses this model to define the levels of osteotomy with special regard to the later surgical margins. According to the planned osteotomy planes and the acquired CT-data not only the custom-made prosthesis but also special osteotomy guides are constructed by the manufacturer to ensure accurate fitting of the prosthesis. The series reported from our institution showed encouraging results (Figure 2) [50]. 

**Figure 3:**
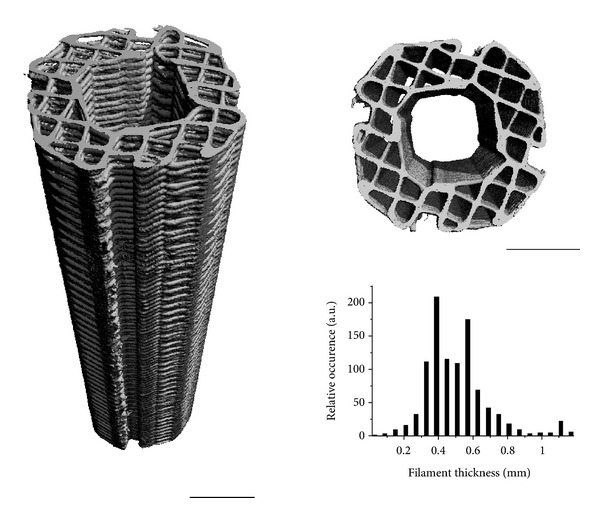
Side and top view of a PCL scaffold for tibia segmental defect regeneration, visualised by microcomputed tomography. The fabrication technique results in scaffolds with well-controlled architecture as evidenced by the narrow filament thickness distribution, leading to a porosity (volume fraction available for tissue ingrowth) of 60%, with interconnected pores. Scale bars are 5 mm.

Although these massive endoprostheses provide orthopaedic oncologists with many reconstructive options, failure rates are still high. Especially in younger patients a reconstructive method would be desirable that does not rely on the use of permanent metal implants but rather on bioactive materials enabling customised reconstruction and supporting natural healing processes. Using computer-aided design (CAD) and fused deposition modelling (FDM) technologies, we are able to produce bioresorbable composite scaffolds fabricated from mPCL, either with or without reinforcement using up to 20 wt%  *β*-tricalcium phosphate (TCP) for bone tissue engineering applications at load-bearing sites (Figure 3). 

**Figure 4:**
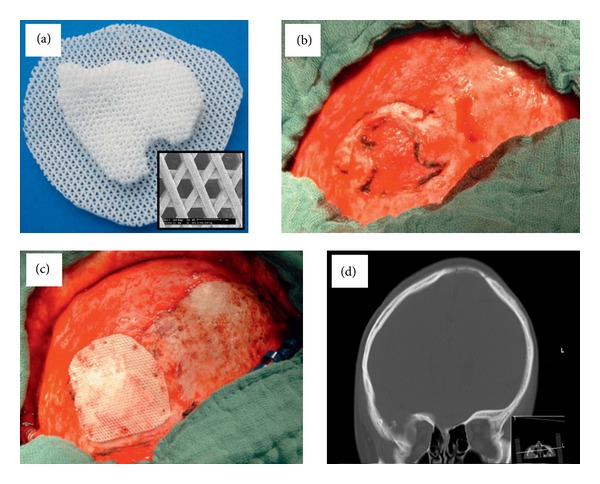
Clinical application of a cell-free polycaprolactone-calcium phosphate scaffold for bone regeneration in a calvarial defect. Scaffold designed using CT imaging data and fabricated by fused deposition modeling (a). Calvarial defect (b). Defect after implantation of the scaffold (c). CT images showing bony consolidation of the defect after 6 months (d). Reproduced with permission from Georg Thieme Verlag (2012) [3].

This scaffold exhibits mechanical and structural properties comparable to cancellous bone and can be specifically adapted to the clinical needs with a fully interconnected pore network structure [45, 51]. A detailed description of the fabrication protocol has been given elsewhere [34, 39, 44]. These scaffolds are already in clinical use and are FDA approved for craniofacial applications [3] (Figure 4).

**Figure 5:**
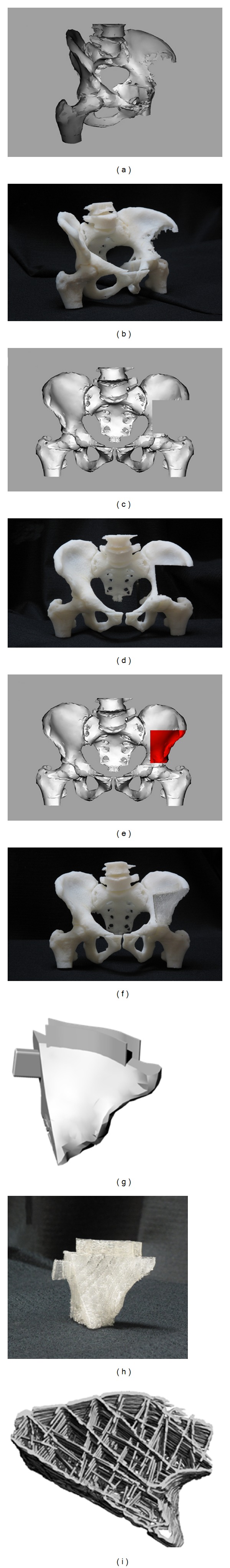
Schematic illustration of the scaffold manufacturing process. A 3D computer-aided designed (CAD) model of the patient's pelvis is fabricated according to data obtained by high-resolution CT ((a), (b)). Using this prototype, the surgeon indicates the osteotomy planes needed to achieve tumour-free resection margins, after which the CAD model is virtually resected ((c), (d)). A scaffold model is then derived by mirroring the healthy side of the pelvis and adjusting the size to fit into the defect ((e), (f)). The scaffold can be armed with flanges or an intramedullary peg to enhance its primary stability ((g), (h)) and exhibits a porous internal architecture to allow for tissue ingrowth and regeneration (i).

In principle, the fabrication process of our scaffolds as depicted in Figure 5 is similar to that described for the patient-specific and individually customised pelvic metal implants. Preoperatively, high-resolution CT data is processed via a 3D medical imaging software (e.g., InVesalius 3.0) supporting the medical DICOM/PACS format to generate a virtual model of the pelvis. In the next step, the data set is converted into a Standard Tessellation Language format (STL) which is the standard format for rapid prototyping applications. Accordingly, a model is built from an acrylonitrile butadiene styrene (ABS) polymer using a 3D fabricator (FDM3000, Stratasys, Eden Prairie, USA) based on fused deposition modeling technology. The model facilitates the haptic perception and orientation both before and during surgery. In close collaboration with the orthopaedic surgeon, the levels of osteotomy are marked in both the virtual and the physical model. Considering the dimensions of the tumor it should be possible to achieve tumor-free resection margins. As it has been previously described for the customised metal implants [50], based on the virtual model, special osteotomy guides can be manufactured to facilitate the later resection and implantation process of the scaffold. As the dimensions of the prospective bony defect are exactly known, the dimensions of the scaffold can be virtually adjusted. Moreover, the form of the scaffold can be adapted to the clinical needs. In the first step, we mirror the healthy side of the pelvis to the affected one to achieve near-physiological conditions. Afterwards the scaffold is armed with flanges and an intramedullary peg to improve its primary stability. Then, Skeinforge software is employed to generate the printing toolpath, which is subsequently modified to introduce porosity allowing tissue ingrowth. An infill density of 0.2 is chosen, corresponding to 80% porosity. Furthermore, the perimeter sections of the toolpath are removed using a custom algorithm to generate open pores to the exterior of the scaffold. According to this modified toolpath the scaffold is then manufactured using again a 3D fabricator. We used a commercially available MakerBot Replicator with poly(D,L-lactide (PDLLA) as biomaterial. PDLLA is a biodegradable thermoplastic polymer which has been successfully applied for fixation in maxillofacial reconstructions before [52, 53] and has been made available for several additive manufacturing techniques such as fused deposition modeling (as in this application) and stereolithography [54]. During surgery, the flanges are fixed with resorbable tacks or screws. Additionally, the contact area between the scaffold and the host bone can be covered with fibrin glue which can serve as a biomimetic template promoting migration of osteogenic cells. 

**Figure 6:**
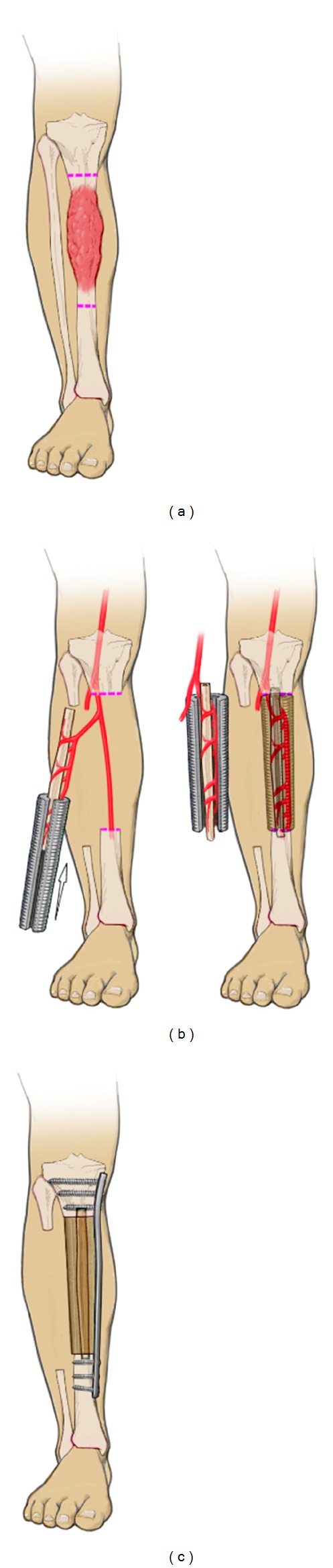
In orthopaedic oncology, vascularised fibula transfer is considered as one of the most suitable techniques for the reconstruction of critically sized defects of the tibia diaphysis due to the mechanical strength, the predictable vascular pedicle, and the hypertrophic potential of the fibula. Combining the autograft with a large bone allograft can enhance the biomechanical properties of the construct. However, the use of allografts can be associated with significant drawbacks such as immunomediated rejection, graft sequestration or transmission of infectious diseases. In addition, the acquisition costs are rather high. A novel biological approach could be to combine an intramedullary fibular autograft with a customised tissue engineered bone construct. After tumor resection (a) a customised mPCL/TCP tube is placed around the vascularised fibula (b) to fill the defect. Together with an internal fixation device, it ensures load distribution and primary stability. Secondary stability is achieved by osseointegration of both the fibula and the porous scaffold (c).

The presented therapy strategy combines the advantages of both CAD/CAM procedures and tissue engineering concepts. Technically, it is not restricted to the application in defects caused by pelvic tumors but can also be transferred to other bony defect sites (Figure 6). 

## 6. Outlook 

Though, small bony defects such as cysts are relatively easy to handle in the routine clinical setting, the management of large defects in load-bearing bones presents a particular challenge in reconstructive surgery and particularly in orthopaedic oncology. In this opinion paper, tissue engineering has been suggested as an alternative strategy to regenerate bone in patients with musculoskeletal sarcoma. We have developed an integrated holistic approach for the reconstruction of bone defects caused by musculoskeletal tumours using patient-specific scaffolds with well-defined macro- and microarchitecture. Though promising case reports have been presented in the literature [3, 55], large clinical studies, which can show the efficacy of this approach in the clinical setting, are still missing. To tackle major bone tissue engineering problems in orthopaedic oncology, researchers have to perform functional assessment of the biological and biomechanical parameters of the regenerated bone. Furthermore, to allow a comparison between different studies, animal models, fixation devices, surgical procedures, and methods of taking measurements need to be standardised to achieve an efficient accumulation of reliable data as a foundation for future developments.
